# Developing a risk framework for assembly construction based on stakeholder theory and structural equation modelling

**DOI:** 10.1371/journal.pone.0301370

**Published:** 2024-05-06

**Authors:** Yin Junjia, Qin Xiaoxiang, Aidi Hizami Alias, Nuzul Azam Haron, Nabilah Abu Bakar

**Affiliations:** 1 Department of Civil Engineering, Faculty of Engineering, Universiti Putra Malaysia, Serdang, Malaysia; 2 Nanning College for Vocational Technology, Nanning, China; Kwame Nkrumah University of Science and Technology, GHANA

## Abstract

Occupational injuries in the construction industry have plagued many countries, and many cases have shown that accidents often occur because of a combination of project participants. Assembled construction (AC) projects have received extensive attention from Chinese scholars as a future trend, but few studies have explored the interrelationships and potential risks of various stakeholders in depth. This study fills this research gap by proposing a multi-stakeholder AC risk framework. The study surveyed 396 stakeholders, then analyzed the collected data and created a risk framework based on Structural Equation Modelling (SEM) and the CRITIC weighting method. The results revealed that factors like "regular supervision is a formality," "blindly approving the wrong safety measures," and "failure to organize effective safety education and training." are vital risks in AC of China. Finally, the study validates the risk factors and the framework with 180 real-life cases, which shows that the proposed framework is theoretically grounded and realistic. The study also suggests multi-level strategies such as introducing AI-based automated risk monitoring, improving the adaptability of normative provisions to technological advances, and advancing the culture of project communities of interest to ensure AC’s safe practices.

## 1. Introduction

**Fig 1:**
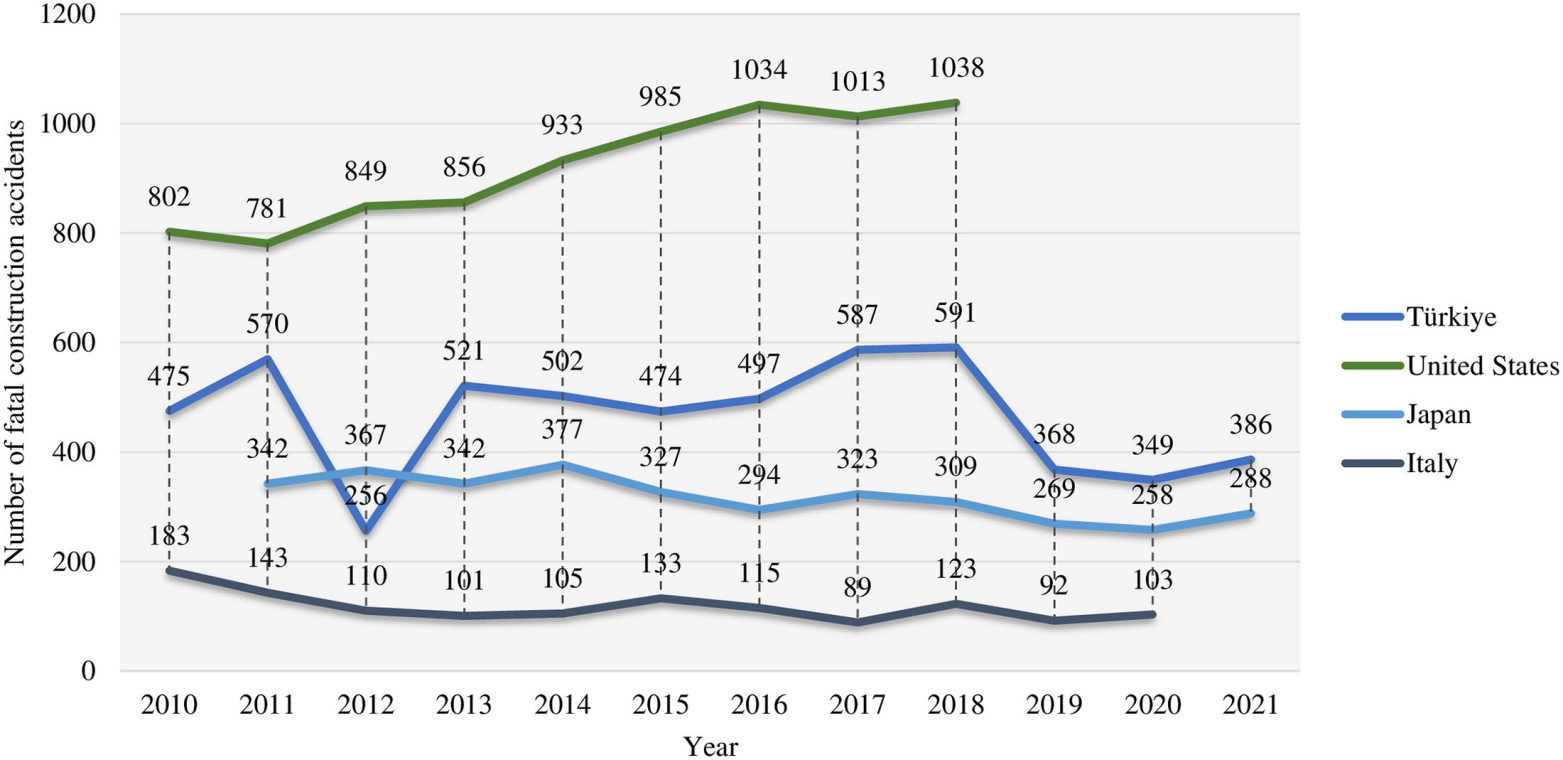
Number of fatal construction accidents from 2010 to 2021.

Due to the complex geology of many construction sites, harsh operating environments, and many unknown factors, construction safety is a problem that plagues many countries [1]. Fig 1 illustrates the number of fatal construction accidents in Turkey, the USA, Japan, and Italy from 2010 to 2021 [2]. Among them, the USA has almost as many fatal construction accidents as the other three countries combined. In 2020, a total of 689 production safety accidents in housing and municipal engineering occurred in China, killing 794 people [3]. Therefore, this topic must be studied further. Standard assembly construction (AC) has five stages: production, transport, storage, hoisting, and installation [4, 5]. In AC, workers are exposed to many hazards, such as falls from heights, scaffolding, unprotected machinery, heavy equipment, and electrocution [6]. In traditional concepts, risk is a combination of possibility and lousy consequences. A practical risk framework is critical for industries and individuals to help them make informed decisions and mitigate potential negative consequences because of increased competition and construction activity [7]. Regrettably, the construction industry in developing countries such as Pakistan and China do not yet have a core risk framework, especially in AC. It consists of several upstream and downstream links that require effective coordination between project participants, including the contractor, consultant, government, and so on, which brings significant challenges to risk management [8]. Each stakeholder’s risk to the project may be affected by their position, knowledge, credibility, actual division of labour, training, and experience. Some scholars have pointed out that 10% of accidents in large-scale mechanical construction are attributable to natural causes, 30% are caused by human error, and 60% are caused by a combination of human error and natural factors [9]. It is inevitable to consider stakeholder dimensions in the risk framework. Unfortunately, existing publications in this area are scarce, and there is a lack of studies that elaborate on the relevance of the various risk stakeholders. In addition, the causal relationship between management responsibilities and stakeholders has not been fully established, which creates barriers to accident recovery and risk allocation. For example, Wang et al. divided construction risks into national, market, and project dimensions, but such a framework can easily ignore operational details [10]; Tserng et al. have also proposed a framework from the knowledge management perspective, emphasizing knowledge accumulation and reuse [11]; Jennifer considered multiple stakeholders in her research, but her macro framework could not be directly applied to the hoisting construction [12]. Many new risks also throw a massive spanner in the works of previous frameworks, such as COVID-19. Construction has the highest rate of COVID-19 infections, which is five times higher than the average for other industries because many tasks require physical contact and intense physical activity for the workers [13]. Therefore, this study aims to develop a risk framework based on a multi-stakeholder perspective to address AC’s rapidly changing occupational safety risks. The objectives of this paper include: (i) To identify potential risks in AC through a literature review and categorize them based on stakeholder theory. (ii) To obtain the opinions of many stakeholders on the risks through survey activities. (iii) To quantify stakeholder correlation using Structural Equation Modelling (SEM) and CRITIC weighting method. (iv) To validate the effectiveness of the risk framework based on real cases.

The remainder of this paper is organized into four sections. Section 2 briefly reviews existing research on the AC risk framework over the past five years. Section 3 describes the research design and methodology. Section 4 sets out the main findings, discusses the significance and limitations of this study, and validates this proposed risk framework with 180 real Chinese accident reports. Section 5 summarises the paper and gives recommendations.

## 2. Previous studies

Assembly construction (AC) is desirable as a future trend in China. The Chinese government has mandated that by 2025, 30% of the country’s annual new construction (by floor area) will be constructed from assembled buildings [14]. Firstly, AC is one of the hallmarks of green development. In this building system, materials such as steel structures are pre-manufactured, with minimal environmental impact and the most efficient use of resources throughout the life cycle, from design to end-of-life disposal [5]. It reduces the generation of construction waste, the use of labor, and the duration of work [15]. Secondly, the future trend is to shift from closed to open systems. ac can develop standardized functional blocks, unify modules, and incorporate personalized integration. It pushes the construction industry toward greater efficiency and environmental friendliness, which comes with many risks. Some of the risks have already been identified in studies. For example, components and materials are usually supplied by suppliers and are prone to traffic congestion, production line failures, or logistical problems. When prefabricated components are arranged to be produced in poorly managed factories, they are prone to quality defects such as dimensional errors, damage to joints, and insufficient load-bearing capacity, leading to assembly difficulties [16, 17]. Since many accidental injuries occur during construction, including "natural disasters" and "man-made disasters," this study focuses on safety risks. Indeed, China has issued specifications on identifying and assessing AC risks to ensure quality and safety in construction. Examples include "Technical Regulations for Assembled Concrete Structures (JGJ1-2014)", "Technical Guidelines for Assembled Steel Modular Buildings," and "Technical Specifications for Safety in Assembled Building Construction (Beijing-Tianjin-Hebei)," "Technical Standard for Assembled Concrete Buildings and Technical Standard for Assembled Wooden Structure Buildings." However, due to the lack of in-depth research on safety risks in AC, these codes cannot effectively guide the various stakeholders on site to fully understand and actively respond to the risks in each link.

Zhou et al. used a social network analysis (SNA) approach to construct a framework for stakeholder-related risks in Hong Kong assembly projects but focussed on productivity performance risks [18]. The study found that poor communication and information exchange between project stakeholders is a significant source of risk. Gong et al. also used social network analysis (SNA) to create a risk network but focused only on AC in less developed regions of China. the study found that AC in these regions is still in the early stages of development, and developers generally lack a dominant role [19]. Hsu et al. developed risk models to capture changes in assembly site requirements for risk aversion but focussed on schedule risk. Common causes of site schedule deviations include inclement weather, late deliveries, labor productivity fluctuations, and crane failures [20]. Through the importance-performance analysis method, Wang et al. found that poor factory management, weak quality systems, large deviations and defects in prefabricated components, and missing parts are the main problems in current AC. However, the study did not result in a defined risk framework [21]. Li et al. combined SEM and system dynamics modeling (SDM) to develop a quality risk model [22]. Ye et al. developed a cost-risk matrix for AC based on the WSR (Wuli-Shili-Renli) model [23]. A systematic analysis of AC project risks in the EPC model from the perspective of a general contractor was carried out by Xia et al. [24]. Chang et al. developed a control model for multi-objective AC to balance the risk and cost. They solved it using the discrete multi-objective particle swarm optimization (discrete-MOPSO) algorithm [25]. The above literature only deals with certain construction processes, specific project organizations, or accident types. There is still a lack of risk studies addressing stakeholders throughout the construction process of AC.

**Fig 2:**
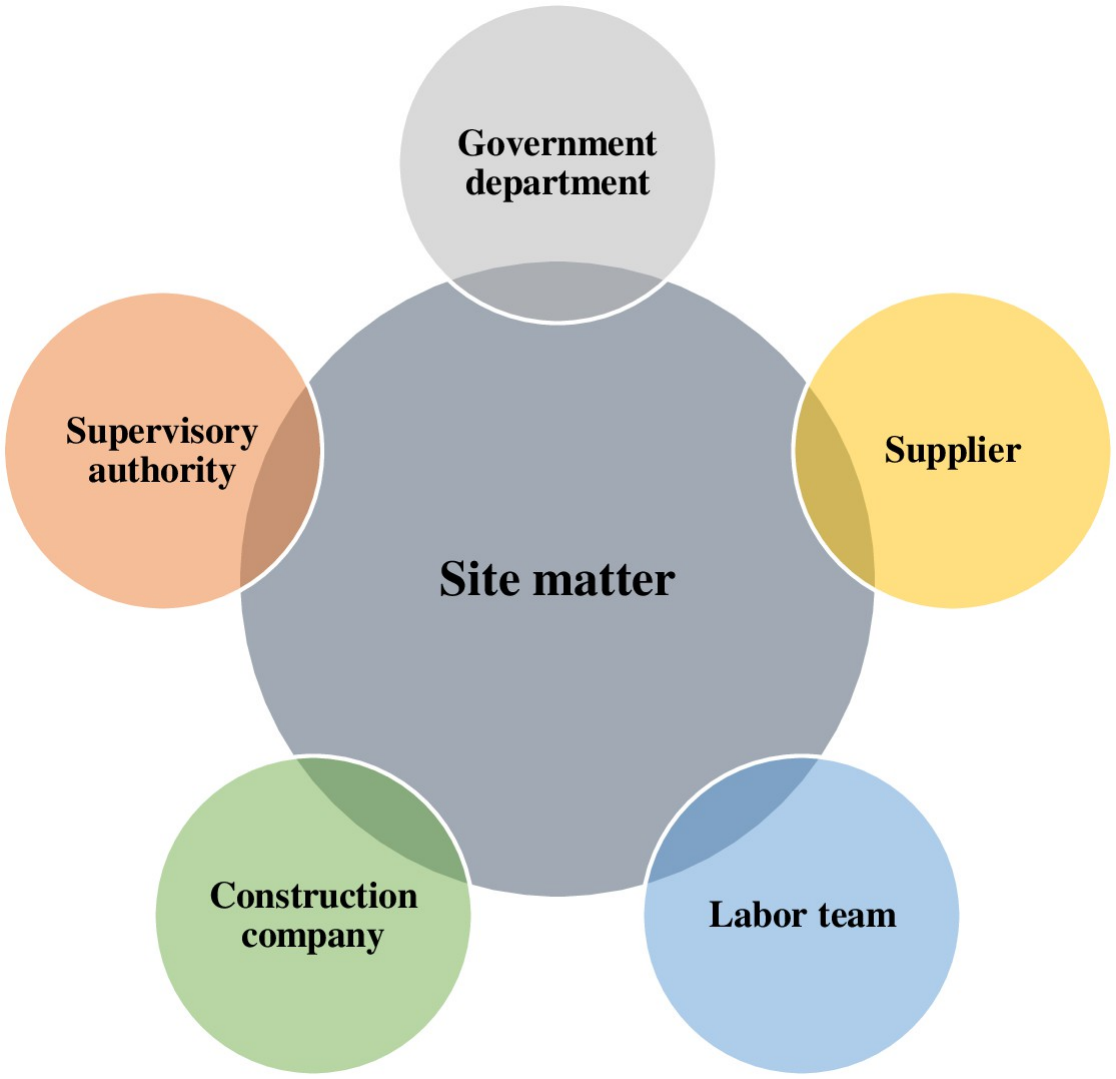
Stakeholders in assembly construction.

Under the traditional definition, risk is the likelihood of an uncertain event or potential loss in each situation [26]. It is usually associated with unpredictable factors, adverse consequences, or unforeseen events. As techniques and technologies evolve, relevant risk maps must be updated to remove uncertainty as new evidence and information become available [27]. To date, there is no adequate theoretical framework for the distribution of safety risk factors in AC, and few studies have explored the causal relationships and interactions between stakeholders in AC. Stakeholder theory is a management theory that argues that organizations should consider all stakeholders, not just one organization, when making decisions and operating [28]. Stakeholders are individuals, groups, or entities that can influence or be influenced by an organization’s actions, policies, and objectives. It is centered on the idea that the success of a project depends on the joint action of all organizations. They usually include shareholders, contractors, employees, suppliers, and government entities [29]. Fig 2 illustrates the relevant stakeholders that affect AC security on site. Therefore, there is a need for a theoretical framework to clarify the safety risks of the stakeholders in AC and to provide a reasonable basis for risk responsibility allocation and accountability. The safety risk framework proposed that synthesizes the perspectives of various stakeholders in AC can be used to guide response strategies.

## 3. Materials and methods

**Fig 3:**
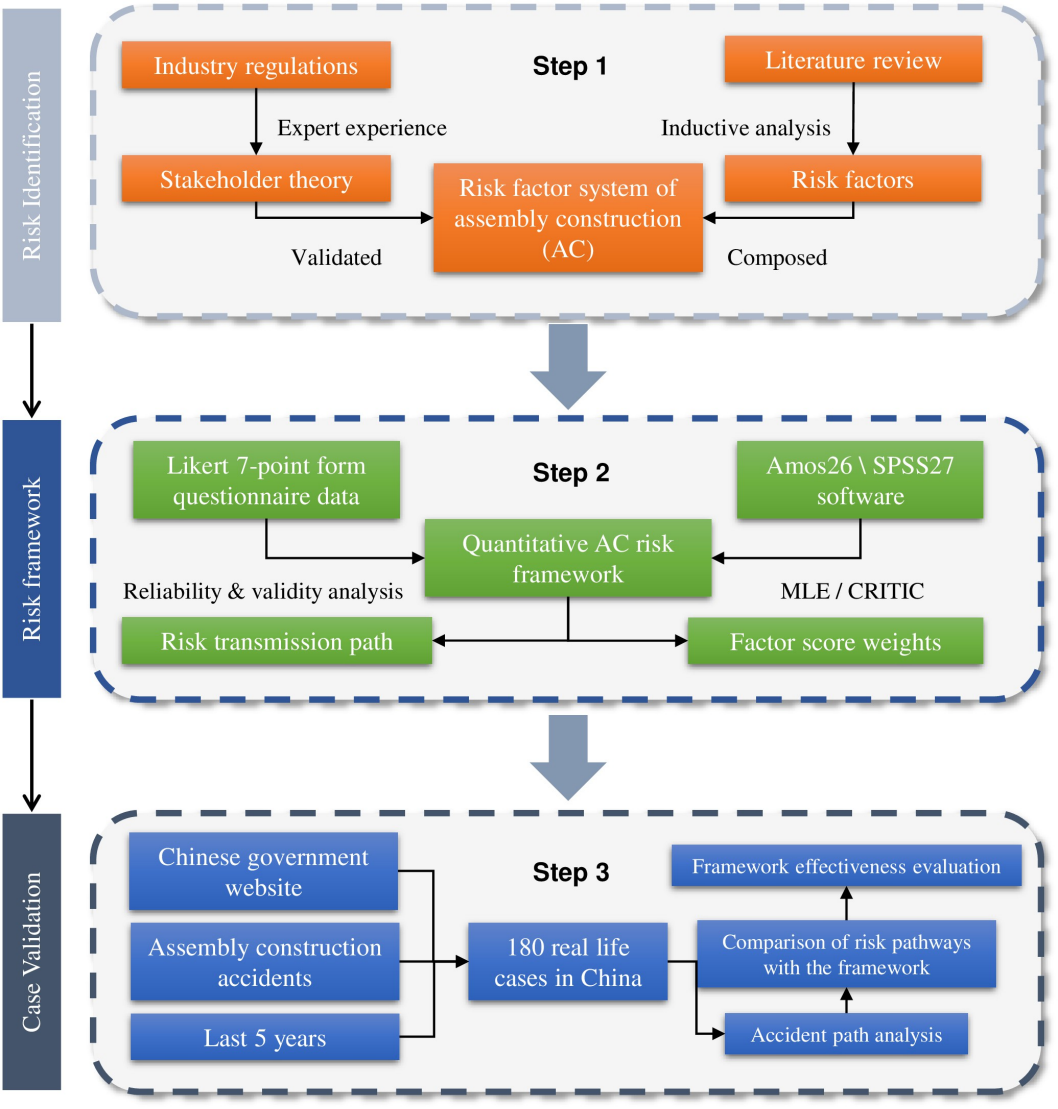
Research flow.

As indicated in Fig 3, this study consists of three main steps: identifying risk factors, constructing a risk framework, and validating the findings.

### 3.1 Risk factor identification

Summarising risk factors by reviewing previous literature is a common and valuable method in research. Identifying and consolidating relevant information about potential risks associated with AC helps to provide a comprehensive understanding of the factors that may contribute to risk and facilitates the next step in risk framing. The study was conducted in the Web of Science (WoS) database through the keywords "prefabricated building," "assembly construction," "safety risk," and "risk factors." Since "prefabricated construction" is a new concept (it began to be widely studied after 2017), there are only 20 articles that are strongly related to safety risks. This study aims to establish a highly applicable framework and excludes studies that adopt specific technologies such as “BIM” and do not consider stakeholders. Finally, this study chose to refine and summarize the risk factors from 12 articles, and eliminated some factors that only appeared once, forming a risk indicator system composed of 35 factors in Table 1.

**Table 1:** Assembly construction risk factors.

*Classifications*	ID	Factors	Source
*Government department (GD)*	G1	Inadequate approval of construction procedures.	[30, 31]
	G2	Laxity in enterprise qualification audit.	
	G3	Large machinery reporting management.	
	G4	Regular supervision is a formality.	
*Supervisory authority (SA)*	SA1	Blindly approving the wrong safety measures.	[32, 33]
	SA2	Confused site supervision system.	
	SA3	Failure to conduct on-site supervision.	
*Construction company (CC)*	C1	Inadequate technical safety briefings.	[34, 35]
	C2	Chaotic arrangement of large machinery on-site.	
	C3	Lack of on-site safety management.	
	C4	Defective construction plans.	
	C5	Failure to organize effective safety education and training.	
	C6	Poor control of material or equipment supply.	
*Site matter (SM)*	SM1	Collapse of buildings and or structures.	[36, 37]
	SM2	Hit by a robotic arm.	
	SM3	Struck by a lifting load.	
	SM4	Crane overturning.	
	SM5	Environmental damage at the construction site.	
	SM6	Electrocution.	
	SM7	Worker falls from height.	
*Labor team (LC)*	L1	Improper driver operation.	[38, 39]
	L2	Improper handling by the rigger.	
	L3	Signaler command failure.	
	L4	Inexperience in construction.	
	L5	Poor health status.	
	L6	Low safety awareness.	
*Supplier (SU)*	SU1	Poor quality of wire ropes.	[40, 41]
	SU2	Low reliability of hooks and reels.	
	SU3	Insufficient load-bearing capacity of wheels or tracks.	
	SU4	Brake insensitivity.	
	SU5	Unstable foundation.	
	SU6	Insufficient operating instructions for machinery.	
	SU7	Poor safety climate in the factory.	
	SU8	Failure to establish proper installation and dismantling procedures.	
	SU9	Poor control of the raw materials used to manufacture the equipment or component.	

**Fig 4:**
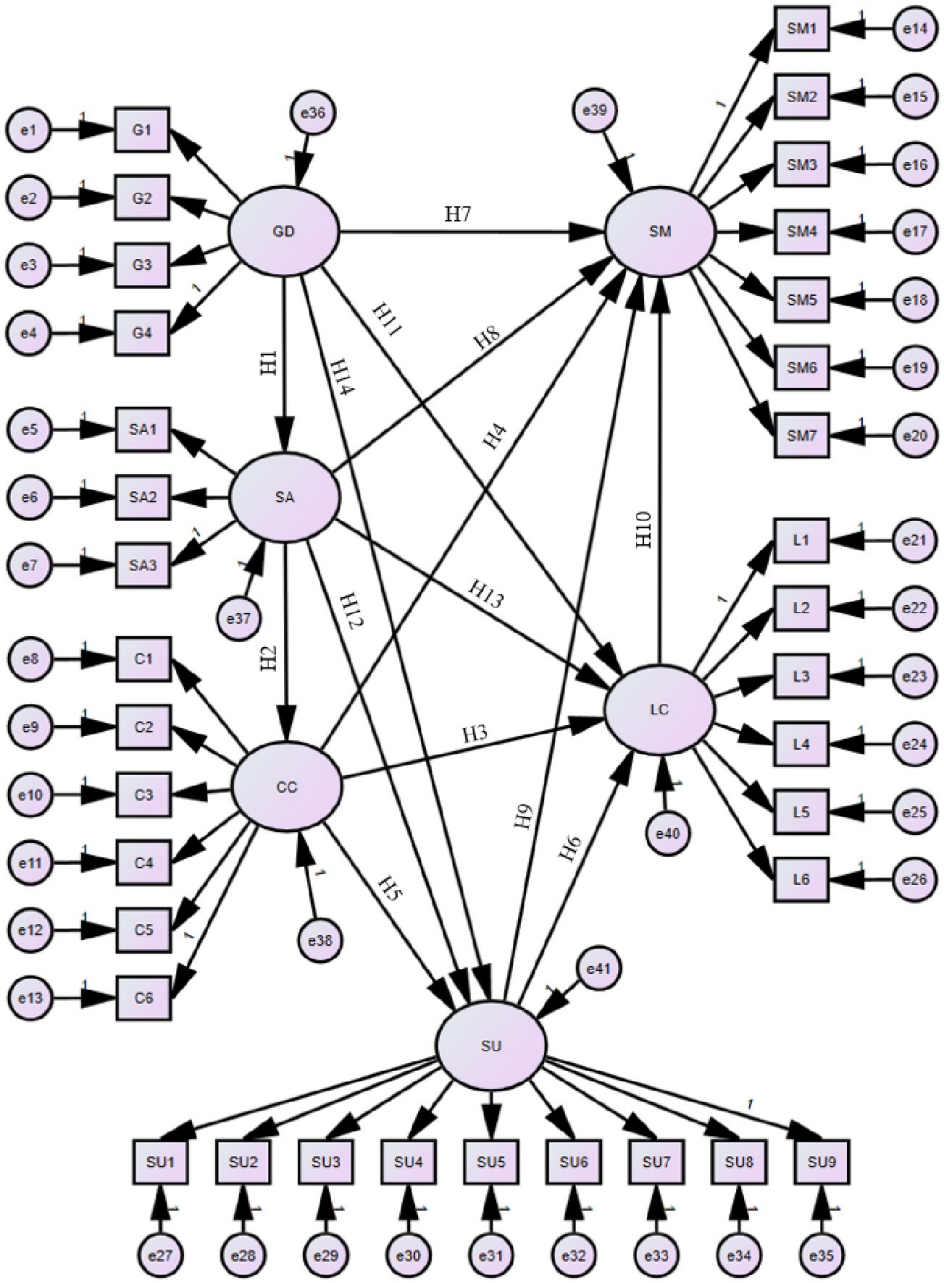
Initial AC risk framework.

The hypothesized model is based on the literature review, which is the basis for testing the relationship between the independent and dependent variables. The developed theoretical model focuses on the relationship between stakeholder risks (e.g., government department, Supervisory authority, construction company), the construction process (e.g., build production, transport, and installation), and the integration of such linkages. As illustrated in Fig 4, the following hypotheses were constructed based on the relationship between risk factors (independent variables) and the build construction process (dependent variables).H1: Government department significantly impacts supervisory authority; H2: Supervisory authority significantly impacts construction company; H3: Construction company significantly impacts labor team; H4: Construction company significantly impacts site matter; H5: Construction company significantly impacts supplier; H6: Supplier significantly impacts labor team; H7: Government department significantly impacts site matter; H8: Supervisory authority significantly impacts site matter; H9: Supplier significantly impacts site matter; H10: Labor team significantly impacts site matter; H11: Government department significantly impacts labor team; H12: Supervisory authority significantly impacts supplier; H13: Supervisory authority significantly impacts labor team; H14: Government department significantly impacts supplier. Significant impact implies that the path is substantial and the Plabel is less than 0.05 [42, 43].

### 3.2 Questionnaire data collection

The survey was administered in China over six months, from September 2023 through February 2024. The questionnaire utilized a 7-point Likert scale to assess the level of all items, ranging from very low to very high values, with levels 1–7 corresponding to very low, low, somewhat low, medium, somewhat high, high, and very high values, respectively. Since this study is exploratory and purposive, it fulfills the requirement of using purposive sampling (or non-random sampling) for questionnaire collection. Purposive means that the questionnaire collection in this study was restricted to "government departments", "supervisory authorities", "construction companies", "labor teams" and "suppliers". This also helps to save limited time and financial cost [44]. Five hundred questionnaires were sent out in this study, and 413 were returned, giving a return rate of 82.6%. Questionnaires with a certain regularity, high consistency, and incomplete answers were excluded, making 396 questionnaires available for subsequent analyses. To develop an SEM, it is recommended that the sample size be at least 200 and at least ten times as large as the observed variables [45]. With 35 observed variables, a sample size 396 was deemed adequate for analysis.

The profile of the respondents of the questionnaire survey, as listed in Table 2. 72.73% of the respondents have a university degree or above; 69.7% are above 30 years old; and the proportion of intermediate and senior engineers reaches 49.75%, which indicates that they have rich experience and exposure in this field. Since many government administrators and front-line experienced workers do not have the relevant professional title of engineer, this study uniformly includes them as others. Therefore, the data source of this study is reliable and lays the foundation for the findings.

**Table 2:** The profile of respondents (N = 396).

Age	Subtotal	Proportions
20–29 years old	120	30.30%
30–39 years old	168	42.42%
40–49 years old	68	17.17%
Over 50 years old	40	10.11%
Academic qualifications	Subtotal	Proportions
High school and below	108	27.27%
Bachelor	244	61.62%
Master	33	8.33%
Doctor	11	2.78%
Title	Subtotal	Proportions
Assistant engineer	170	42.93%
Mid-level engineer	157	39.65%
Senior engineer	40	10.10%
Other	29	7.32%
Working experience (years)	Subtotal	Proportions
1–5	68	17.17%
5–10	91	22.98%
10–15	139	35.10%
More than 15	98	24.75%
Working sector	Subtotal	Proportions
Government department	56	14.13%
Supervisory authority	54	13.64%
Construction company	130	32.83%
Labor team	70	17.68%
Supplier	86	21.72%

Before analyzing questionnaire responses, it is essential to undertake reliability and validity tests to ensure the data’s robustness. In this regard, Cronbach’s alpha test is a widely used measure of questionnaire reliability that assesses the degree of internal consistency among the items of a questionnaire. Cronbach’s alpha is at least 0.70; the closer it is to 1, the higher the confidence in the data [46]. We used SPSS27^®^ for reliability analysis. The findings presented in Table 3 reveal that Cronbach’s α for all the latent variables ranged from 0.887 to 0.958, which surpasses 0.7. As such, the factors under examination exhibit commendable internal consistency.

**Table 3:** Variable reliability index value.

Sample	Number of questions	Cronbach’s α
Overall	35	0.957
Latent variable	Total observed variables	Cronbach’s α
Government department	4	0.920
Supervisory authority	3	0.887
Construction company	6	0.924
Site management	7	0.934
Labor team	6	0.930
Supplier	9	0.958

To ensure the validity of the questionnaire, two statistical tests, namely, the Kaiser—Meyer—Olkin (KMO) test and Bartlett’s test of sphericity, are recommended for use. The KMO coefficient should be at least 0.8 [47]. Bartlett’s test of sphericity measures the correlation between variables, with a coefficient of less than 0.01 considered desirable. The KMO coefficient of this study was calculated to be 0.961, indicating that the data were adequately distributed. Moreover, Bartlett’s test resulted in a value of 0.000, which signifies a significant level of correlation between variables. Therefore, the collected data can be regarded as reliable and suitable for SEM.

### 3.3 Structural equation modelling

Structural equation modelling (SEM) is a statistical technique for exploring complex relationships among multiple variables in various disciplines. Kassem used PLS-SEM for risk management assessment of oil and gas construction projects [48]. SEM combines factor analysis and regression analysis and allows researchers to examine both measured observed (measured) and latent (unobserved) variables. The following are the basic formulas for SEM [49]:

#### (1) Measurement equation

Suppose we have a latent variable (e.g., job satisfaction) that is not directly observable but can be indirectly measured by multiple indicators (e.g., scale questions in a risk factor importance questionnaire). The measurement equation describes the relationship between the latent variable and the indicators. Typically, it takes the form of a linear regression model as follows:

X=Λη+ϵ (1)

where:

*X* is the indicator vector.

Λ is the factor loading matrix, representing the relationship between the indicator and the latent variable.

*η* is the vector of latent variables.

*ϵ* is the measurement error vector.

#### (2) Structural equation

A structural equation describes the relationship between latent variables. It usually includes multiple latent variables and direct and indirect effects between them. A simple structural equation can be expressed as:

$$\eta_{1}=\beta_{21}\eta_{2}+\beta_{31}\eta_{3}+\zeta_{1}$$ (2)

Where:

*η*_1_ is a latent variable.

*β*_21_ and *β*_31_ are path coefficients that indicate the latent variables’ relationship.

*ζ*_1_ is the structural error.

Maximum Likelihood Estimation (MLE) is a frequency school parameter estimation method for determining the parameters of a probability distribution given the observed data [50]. It computes an estimate of a parameter by maximizing a likelihood function that maximizes the probability that the observed data will be unbiased, stable, valid, and asymptotically generally distributed given that parameter [51]. This study uses MLE to estimate the parameters in the model, such as path coefficients, factor loadings, and error variance.

### 3.4 CRITIC weighting method

The CRITIC weighting method is an objective assignment method, compared to the assignment of AHP, the preferential graph method, the entropy value method (EVM), and the grey correlation method; it makes use of the volatility of the data and the correlation relationship situation [52]. It is not that the bigger the number, the more important; it is entirely using the data’s objective attributes. Before CRITIC analysis, the data must be quantified, and forward or reverse quantification is generally recommended. Its calculation steps are as follows [53, 54]:

1. Assume that there are n samples to be evaluated and p evaluation indicators, forming the original indicator data matrix *X*.
2. Dimensionless processing: The indicators are dimensionless to eliminate the influence on the evaluation results due to the different scales. If the indicator’s value is more significant, the better (positive indicator), use the positive processing. If the smaller the value of the indicator, the better (reverse indicator), use the reverse treatment.
3. Calculation of indicator variability: expressed in standard deviation, the larger the standard deviation, the greater the difference in the indicator’s value, the more weight should be assigned.
4. Calculating Indicator Conflict: expressed as a correlation coefficient, the stronger the correlation, the less conflict and weight should be assigned.
5. Calculate the information content: indicate the size of the role of the indicator in the whole evaluation index system; the more information content, the more weight should be assigned.
6. Calculation of objective weights: The final objective weights are obtained considering the variability and conflict of the indicators.

## 4. Result and discussion

### 4.1 Risk framework

**Fig 5:**
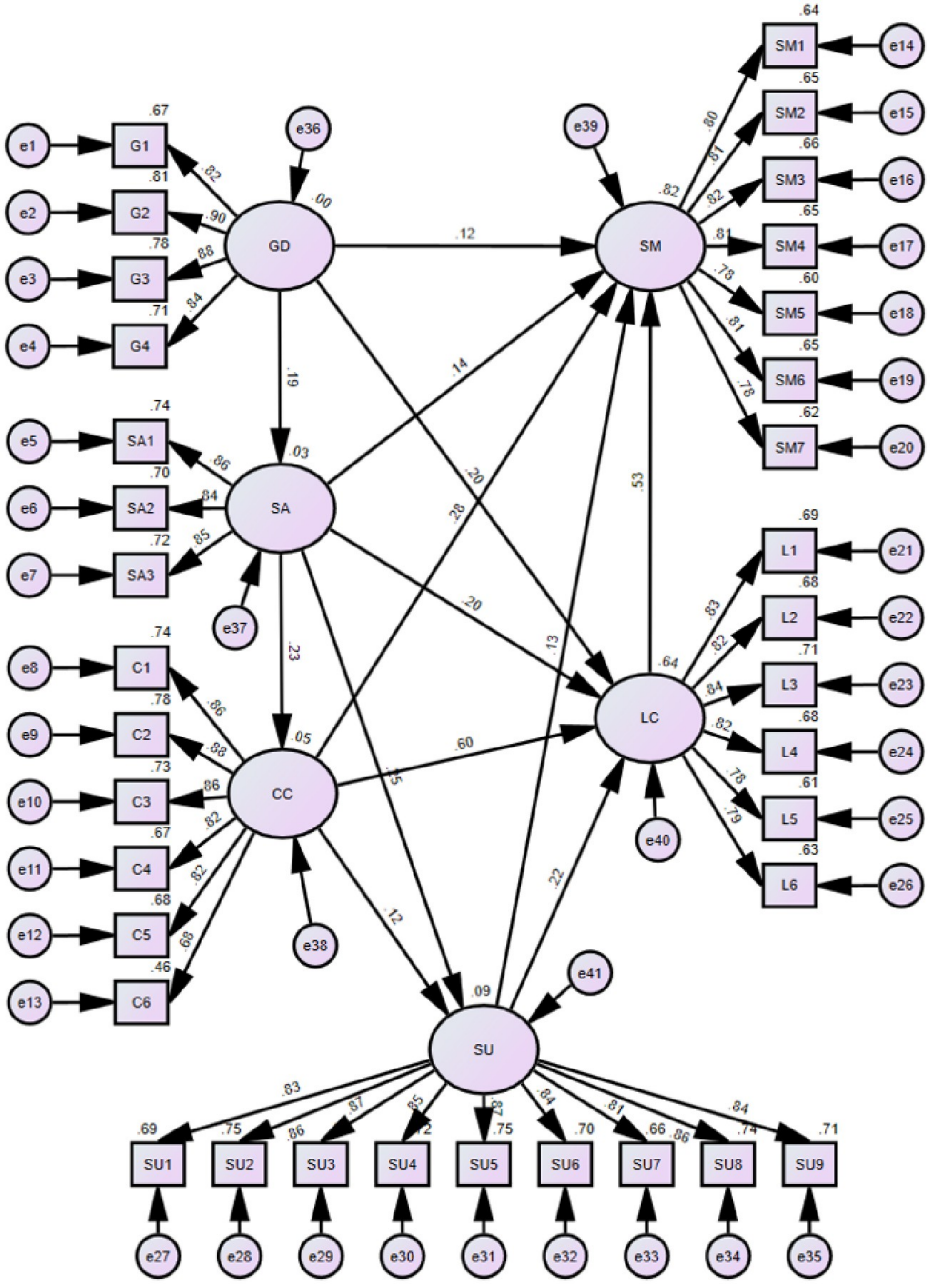
Final AC risk framework.

Following the stated hypotheses, we constructed an SEM framework utilizing AMOS26^®^. The initial framework can be divided into two parts, one for the measurement model and one for the structural model, as shown in Fig 5. The six dimensions of risk (latent variables) are measured indirectly through their respective observed variables, sub-risks of different dimensions are transmitted through interdimensional interactions. Furthermore, all the hypotheses were validated except H14. According to our interviews with experts, many believe that the government’s role to suppliers is to issue operating licenses and control market access rather than directly affecting on-site transactions of equipment and materials, which are indirectly regulated in the AC. For the hypotheses to be considered significant, the Plabel value should be less than 0.05. In this case, the Plabel values are ***, which suggests that the effects are substantial and that the regression coefficients are not zero. The model’s result is the total effect, including direct and indirect effects. The direct effect measures the influence of the cause variable on the outcome variable. In contrast, the indirect effect measures the combined impact of each dependent variable on the outcome variable. This is determined by summing the products of the path coefficients. A detailed breakdown of these effects can be found in Table 4.

**Table 4:** Total effects of the final model.

Path			Standardized estimate	S.E.	C.R.	Plabel
SA	<---	GD	0.186	0.052	3.367	***
CC	<---	SA	0.227	0.034	4.066	***
SU	<---	CC	0.123	0.093	2.301	0.021
SU	<---	SA	0.248	0.059	4.502	***
LC	<---	SU	0.22	0.037	5.693	***
LC	<---	CC	0.599	0.088	11.283	***
LC	<---	SA	0.204	0.042	4.97	***
LC	<---	GD	0.197	0.036	5.225	***
SM	<---	SA	0.137	0.032	3.994	***
SM	<---	LC	0.533	0.053	8.987	***
SM	<---	GD	0.122	0.027	3.861	***
SM	<---	CC	0.276	0.072	5.733	***
SM	<---	SU	0.128	0.028	3.973	***
G4	<---	GD	0.845			
G3	<---	GD	0.884	0.048	22.437	***
G2	<---	GD	0.9	0.048	23.055	***
G1	<---	GD	0.821	0.051	19.922	***
SA3	<---	SA	0.85			
SA2	<---	SA	0.839	0.052	19.335	***
SA1	<---	SA	0.862	0.05	19.866	***
C6	<---	CC	0.681			
C5	<---	CC	0.824	0.112	14.999	***
C4	<---	CC	0.817	0.114	14.881	***
C3	<---	CC	0.856	0.112	15.516	***
C2	<---	CC	0.882	0.119	15.923	***
C1	<---	CC	0.859	0.112	15.556	***
SM1	<---	SM	0.8			
SM2	<---	SM	0.809	0.055	18.313	***
SM3	<---	SM	0.815	0.056	18.511	***
SM4	<---	SM	0.808	0.056	18.296	***
SM5	<---	SM	0.777	0.055	17.337	***
SM6	<---	SM	0.809	0.056	18.319	***
SM7	<---	SM	0.784	0.057	17.568	***
L1	<---	LC	0.83			
L2	<---	LC	0.824	0.048	19.743	***
L3	<---	LC	0.84	0.049	20.362	***
L4	<---	LC	0.823	0.049	19.731	***
L5	<---	LC	0.783	0.048	18.286	***
L6	<---	LC	0.791	0.049	18.578	***
SU9	<---	SU	0.841			
SU8	<---	SU	0.859	0.044	22.097	***
SU7	<---	SU	0.811	0.048	20.06	***
SU6	<---	SU	0.837	0.046	21.1	***
SU5	<---	SU	0.866	0.045	22.408	***
SU4	<---	SU	0.85	0.045	21.665	***
SU3	<---	SU	0.871	0.046	22.637	***
SU2	<---	SU	0.864	0.045	22.307	***
SU1	<---	SU	0.832	0.046	20.92	***

The evaluation metrics for the model fit are demonstrated in Table 5. The overall model fit is perfect.

**Table 5:** SEM fit summary.

Fit Index	Criteria	Value	Results	Reference
CMIN/DF	< 2.0, excellent fit	1.502	Nice	[55–57]
GFI	> 0.8, acceptable	0.899	Nice	
	> 0.9, excellent fit			
AGFI	> 0.8, acceptable	0.884	Acceptable	
	> 0.9, excellent fit			
IFI	> 0.9, excellent fit	0.977	Nice	
TLI	> 0.9, excellent fit	0.975	Nice	
CFI	> 0.9, excellent fit	0.977	Nice	
RMSEA	< 0.05, excellent fit	0.036	Nice	

### 4.2 Ranking of risk factors

CRITIC weights are calculated using the variability of evaluation indicators and the conflict between evaluation indicators as criteria (See Table 6). Indicator variability is measured using the standard deviation; the higher the standard deviation, the higher the weight; conflict is measured using the correlation coefficient between indicators; the more robust the correlation between indicators, the lower the conflict, and the lower the weight. Informativeness is calculated as the product between indicator variability and conflict indicators.

**Table 6:** CRITIC weighting results.

Item	Mean	Indicator variability	Conflict of indicators	Volume of information	Weights
G1	4.23	2.096	22.168	46.475	3.30%
G2	4.27	2.071	21.249	44.006	3.12%
G3	4.394	2.059	21.702	44.684	3.17%
G4	4.316	1.991	23.541	46.867	3.32%
SA1	4.851	1.834	25.697	47.12	3.34%
SA2	4.871	1.9	25.289	48.057	3.41%
SA3	4.907	1.858	25.358	47.114	3.34%
C1	4.447	1.968	20.84	41.013	2.91%
C2	4.52	2.078	19.815	41.166	2.92%
C3	4.528	1.961	21.041	41.257	2.93%
C4	4.442	2.014	20.993	42.273	3.00%
C5	4.477	1.972	20.448	40.334	2.86%
C6	3.654	1.421	21.061	29.928	2.12%
SM1	4.747	1.895	17.849	33.828	2.40%
SM2	4.745	1.874	17.881	33.509	2.38%
SM3	4.732	1.943	17.632	34.264	2.43%
SM4	4.644	1.922	17.854	34.311	2.43%
SM5	4.745	1.854	18.137	33.619	2.38%
SM6	4.816	1.913	17.689	33.839	2.40%
SM7	4.773	1.922	18.172	34.932	2.48%
L1	4.578	2.019	18.068	36.484	2.59%
L2	4.699	1.915	17.942	34.366	2.44%
L3	4.662	1.985	17.681	35.1	2.49%
L4	4.576	1.983	18.264	36.211	2.57%
L5	4.563	1.878	18.743	35.203	2.50%
L6	4.674	1.938	18.796	36.419	2.58%
SU1	4.611	1.96	22.693	44.488	3.16%
SU2	4.649	1.981	22.777	45.123	3.20%
SU3	4.652	2.025	22.125	44.805	3.18%
SU4	4.576	1.95	22.289	43.475	3.08%
SU5	4.561	1.955	22.013	43.033	3.05%
SU6	4.672	1.947	21.982	42.797	3.04%
SU7	4.611	2.018	22.951	46.309	3.28%
SU8	4.667	1.911	21.815	41.68	2.96%
SU9	4.593	2.01	22.7	45.622	3.24%

**Fig 6:**
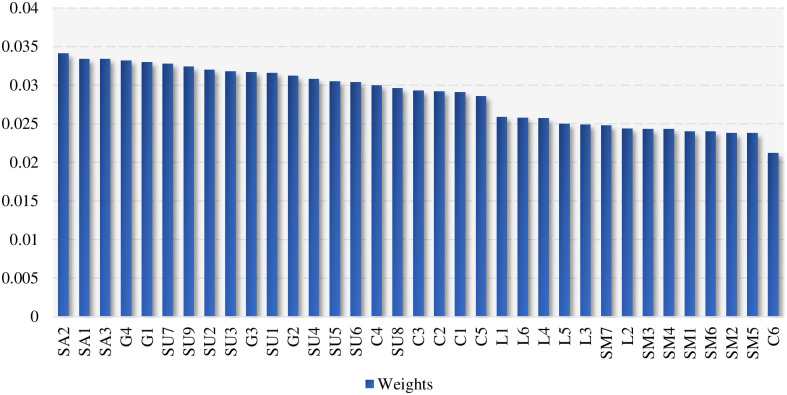
Ranking the importance of AC risk factors.

The final ranking of weights (see Fig 6) is calculated by normalizing the volume of information.

The Government Risk Factor emphasizes the importance of strict regulation, thorough review processes, and active oversight in AC. "Regular supervision is a formality" is the most severe issue. Regular inspections are critical to identify and address deviations from approved construction procedures, ensure compliance with safety standards, and maintain quality control. If supervision is a mere formality and lacks real oversight, it increases the likelihood of construction errors and safety violations. The second is "inadequate approval of construction procedures." Assembled buildings need to follow a series of approval procedures, including design approval and construction permits. Ensuring the assembled building’s safety, integrity, and compliance is critical. If the approval process is inadequate, it may allow non-compliant practices or shortcuts, which may affect the structural stability and safety of the assembled building. The third is "large machinery reporting management," which aims to ensure that large machinery, such as cranes, is within a reasonable working life and to prevent end-of-life machinery from re-entering the market. Lastly, there is "laxity in enterprise qualification audit." Qualified enterprises must undertake assembled buildings. However, suppose the Government is not stringent in qualification vetting. In that case, it may lead to the participation of enterprises that do not have sufficient experience and technical capability, thus affecting the quality and safety of the work.

Supervisory authorities must strengthen professional training, establish an effective supervision system, and strictly enforce on-site supervision duties. The most considerable risk is a "confused site supervision system." A clear and effective supervision system is essential in construction. If the site supervision system is confusing, it will create ambiguity in roles, responsibilities, and safety procedures. This confusion can compromise the implementation of safety measures, hinder emergency response, and lead to an unsafe work environment. The second is "blindly approving the wrong safety measures." Supervisory bodies play a critical role in reviewing and approving safety measures to ensure that they comply with industry standards and are appropriate for a specific assembly building project. A lack of expertise or careful review may result in the implementation of ineffective or incorrect safety measures, putting workers and the construction site at risk. Finally, there is the "failure to conduct on-site supervision". Supervisory agencies should conduct regular site inspections and take side visits to check the progress, quality, and safety of the construction work on the more dangerous processes. If the supervisory body does not conduct on-site supervision, it creates a supervisory vacuum, leaving safety violations or problems unchecked for a long time.

Safety risks in AC require effective management by construction companies from several perspectives, including implementing robust safety protocols, providing adequate training, effectively organizing the construction site, and maintaining supply chain control. The most significant risk is "defective construction plans." The construction program is the blueprint for the project. If they are flawed or incomplete, they can lead to construction errors, delays, and safety issues. Next is "lack of site safety management." Effective on-site safety management includes continuous monitoring, hazard identification, and implementation of safety measures. The safety risk will increase significantly if a construction company lacks professional safety management personnel and safety signs. Thirdly, "chaotic arrangement of large machinery on-site." AC usually involves large machinery and equipment, such as material hoists and tower cranes. If the layout of machinery and equipment is chaotic, it will impede the construction process, increase the likelihood of collisions or accidents, and create an unsafe working environment. The fourth is "inadequate technical safety briefings." Unclear technical briefings may lead to an inadequate understanding of construction processes, safety practices, and risk control measures by construction personnel, increasing the risk of construction accidents. The fifth is "failure to organize effective safety education and training." Workers must understand the characteristics of AC, safety practices, and emergency response methods. If there is a lack of adequate safety education and training, construction workers may not use the equipment properly or be able to cope with emergencies. Finally, there is "poor control of material or equipment supply." AC requires large quantities of prefabricated components and materials. Lack of control over the supply chain can lead to delays, shortages, or the use of substandard materials, affecting construction quality and safety.

Addressing the risk factors in the labor force requires promoting a strong safety culture and ensuring that labor team members are well-equipped and aware of safety procedures. The most considerable risk is "improper driver operation." Labor team members operating vehicles or heavy equipment must be trained appropriately to ensure safe and efficient operation. If the driver lacks the necessary skills or operates improperly, the safety of the labor team and others on the construction site is at risk. The second is "low safety awareness". A culture of poor safety awareness can lead to labor team members ignoring safety procedures, misusing equipment, and ignoring potential hazards. Third is "inexperience in construction". Assembled building construction standards are high, and the precision of components is stringent. Lack of sufficient construction experience will lead to improper operation, quality problems, or safety hazards. Fourth is "poor health status". Health problems can affect the ability of workers to perform their tasks safely and effectively. Poor health labor team members may suffer from poor concentration, coordination, or physical fitness, leading to an increased risk of accidents on the construction site. The fifth is "signaller command failure." In lifting operations, the signaller is responsible for directing the movements of the lifting machinery. Failure to direct can lead to dangerous situations such as collision or instability of components. Lastly, there is "improper handling by the rigger." Lifting is critical to assembly building construction. If the rigger is not operating correctly, it may result in falling, tilting, or damage to the components.

Addressing these risk factors for suppliers requires comprehensive quality control measures, adherence to safety standards during manufacturing, and ensuring that equipment components meet the specifications required for assembly and construction. The most considerable risk is a "poor safety climate in the factory." A lack of attention to safety in the factory can lead to substandard manufacturing practices, leading to safety risks in the supplied equipment. Next is "poor control of the raw materials used to manufacture the equipment or component." The quality of the raw materials used in production directly impacts the final product’s reliability and safety. Poor control of raw materials can lead to product defects. The third is the "low reliability of hooks and reels." Hooks and reels are critical for carrying material. If unreliable, they can cause material to fall and equipment to fail. The fourth is "insufficient load-bearing capacity of wheels or tracks." In AC, mobile equipment (e.g., cranes and winches) needs to run on tracks. If the track or wheels have insufficient load-bearing capacity, it may lead to equipment instability. The fifth is "poor quality of wire ropes." Wire rope is a critical component in various lifting and rigging applications. Poor quality ropes may have manufacturing defects, be weak, or more prone to wear and tear, and if they are not of the required quality, they can lead to breakage, corrosion, or other problems. The Sixth is "brake insensitivity." Brakes are used to control the movement of equipment. If the brake is not sensitive, it will lead to the equipment being unable to stop accurately, loss of control, and collision. Seventh is an "unstable foundation". An unstable foundation can cause equipment to sway, tilt, or collapse. The eighth is "insufficient operating instructions for machinery." A lack of operating instructions can lead to misuse. Lastly, there is a "failure to establish proper installation and dismantling procedures." Large machinery must follow strict installation and dismantling procedures. If the process is not correct, it can lead to damage to the equipment.

### 4.3 Case validation

**Fig 7:**
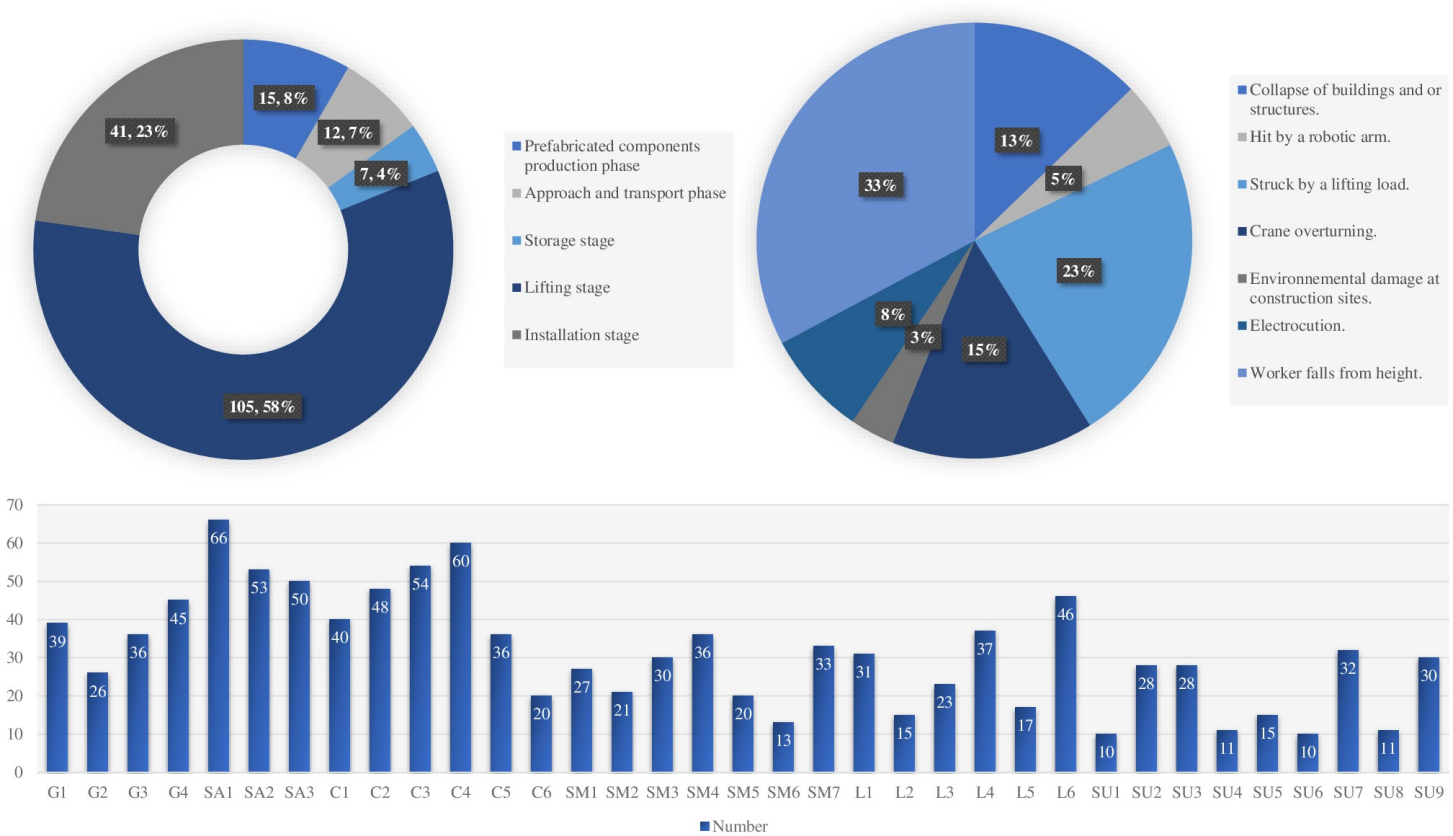
Results of case analysis.

As illustrated in Fig 7, the risk factors proposed in this paper were validated with 180 accident cases related to 2018–2024 on the Chinese government website. The causes of the accidents were counted, and each accident report was analyzed based on the proposed AC risk framework. The study found that several critical stages in the assembly building construction process are prone to accidents. (1) Prefabricated component production stage: When manufacturing prefabricated components, material defects, artistry problems, or operational errors may occur, leading to safety risks, but this stage accounts for a relatively low number of accidents. (2) Inbound and transport phase: When transporting and handling prefabricated components, traffic accidents, dumping of components, or improper loading and unloading may occur, resulting in injuries. The risk at this stage is high. (3) Storage phase: During storage of precast components, slipping of components, improper stacking or unstable storage areas may occur. Accidents at this stage are also relatively low. (4) Lifting stage: When lifting prefabricated components, instability of the spreader, swaying of the components, or failure of the lifting equipment may occur, resulting in serious accidents. This phase carries the highest risk. (5) Installation stage: When installing prefabricated components into a building, improper connections, detachment of components, or insecure installation may occur. This stage also has a high percentage of accidents. In addition, the percentage of accidents involving falls from height is the highest. This is mainly because AC usually involves much work at height. This is followed by object strike accidents, which account for 23% of the total. Crane injuries also accounted for a high percentage of accidents, including injuries from mechanical arms as well as equipment tipping over. Other accidents, such as electrocution, structural collapse of buildings, and damage to the construction environment, also deserve more attention from practitioners.

From these, this paper has selected five real-life cases as representatives to test the real-world applicability of the proposed risk framework, as depicted in Table 7. It provides evidence that the framework is theoretical and has practical utility.

**Table 7:** Accident path verification.

No.	Description	Risk pathway
1	The construction company transported a ZYJ800B hydraulic static pile driver to the south-west corner of the site, because of which the wire rope was detached from the hook under horizontal tension, causing the pile driver to fall and hit the crane, resulting in the death of one person.	C5-»L1-»SM3
2	While lifting the load-bearing steel beam, the northern end of the beam, which was not secured, fell from its mounting position to the ground, causing the southern end to rise. The deceased lost his center of gravity due to the small standing position (dangerous position) and the fact that he was holding on to the load-bearing steel beam and fell from a position of about 6 meters from the ground.	SU7-»L1-»SM7
3	The boom of the truck crane was undergoing clockwise slewing (swinging of the boom from directly behind to the left rear), and when the boom slewed to a point near the top of the left rear outrigger, the crane involved in the incident became unstable and tilted over on its left side. The concrete underneath the left rear outrigger was subjected to a breaking force and sank.	C3-»SU6-»L1-»SM4
4	The formwork was lifted without being secured. After the formwork was lifted onto the vehicle, the worker climbed to the top of the formwork to remove the hook, causing the person and the formwork to tip and slide to either side of the vehicle, killing the worker.	SU8-»L2-»SM3
5	During the downward movement of the counterweight No. C2 was to be placed in position, but the first section of the lifting arm suddenly broke, the counterbalance arm rotated and fell, and it reversed on the ground. The installer and the crane driver fell to the ground at about 19.5 meters.	SA3-»C1-»SU9-»L4-»SM7

Note: The cases in this table are taken from https://www.gov.cn/.

The data shows that accidents occur mainly in lifting and component installation, involving special procedures and conditions. The most prominent risk subject is the supervisory bodies because many rely on grey income, which leads to formalism in the entry of components and the approval of processes and lays the fuse for the development of accidents. The results of analysing AC accidents in the database are primarily consistent with the results of the risk framework, thus confirming the validity of the proposed model.

### 4.4 Discussion

Multi-stakeholder theory provides a more comprehensive dimension for understanding construction risk by incorporating the perspectives of different stakeholders, such as contractors, regulators and project owners. It has become a hot topic pursued by many scholars [58, 59]. Compared with other studies, diverse perspectives ensure the framework captures a broader range of expected and unforeseen potential risks and facilitates effective communication and collaboration among stakeholders [60, 61]. This inclusiveness also enhances the adaptability of the framework to different project contexts (e.g. power facilities, bridges and tunnels) and application environments. There are also limitations to this study, as its generalizability may be restricted since it takes a China-wide sample, and the Chinese construction industry has unique contextual factors and regulatory frameworks. Therefore, future studies could incorporate samples from different countries or regions, considering local regulations, economic conditions and industry standards. However, this does not prevent the methodology of this study and the associated risk factors from being informative for studies in other regions. In addition, future research should attempt to integrate multiple emerging technologies such as sensors, IoT, and generative artificial intelligence such as ChatGPT4 to continuously track critical parameters during construction and provide real-time text reports.

## 5. Conclusion

Assembled buildings as a future trend can enhance the overall sustainability of human settlements, but they also pose safety hazards to the construction industry. Effective risk management requires a joint response from various stakeholders, so a clear risk framework is fundamental. This paper aims to identify and rank important risk factors affecting AC and then construct a quantitative risk framework. First, 35 essential risk factors were summarized by reviewing previous papers and after the expert talk. Secondly, 396 practitioners were surveyed by anonymous questionnaires, and the SEM-based risk framework was constructed based on the multi-stakeholder perspective and directive relationship, and the relationship between them was identified. Then, the risk factors were ranked with the help of the CRITIC weighting method to identify the critical risk factors. Finally, the proposed risk factors and framework are validated through 180 real cases. The results show that in China, supervisory organizations have the most significant influence on safety management and are the direct defenders of political regulations. Risk factors such as "Blindly approving the wrong safety measures," "Confused site supervision system," and "Failure to conduct on-site supervision." are at the top of the list. supervision" are the most significant problems they face. "Regular supervision is a formality" of the government departments, "Poor safety climate in the factory" of the suppliers, "Defective construction plans" of the construction companies, and "Improper driver operation" of the labor team are the most significant risk factors for each. Therefore, there is a need to foster a strong risk community of destiny on projects, including a collective commitment to safety by all stakeholders, regular regulatory audits and campaigns, standardized procedures, and checklists.

## Supporting information

S1 Data (XLSX)

S1 File (PDF)
