# Supplementary material for: Developing a risk framework for assembly construction based on stakeholder theory and structural equation modelling
Source: PLoS One. 2024 May 6;19(5):e0301370. doi: 10.1371/journal.pone.0301370 (PMC11073704; doi:10.1371/journal.pone.0301370)
Supplement: S1 File — (PDF) [file pone.0301370.s002.pdf]

QUESTIONNAIRE ON SAFETY RISK FACTORS FOR ASSEMBLY CONSTRUCTION

Dear Sir/Madam,

I invite you to participate in an academic research survey on potential risk factors affecting safety in China's assembly construction. This is crucial to promoting the healthy and sustainable development of China's construction industry. Therefore, please complete the questionnaire based on your project experience, ensuring you do not disclose commercial or confidential information.

This study is voluntary, and you may discontinue answering at any time. Please note that your responses will be kept strictly confidential. This study does not involve any economic and ethical conflicts. In addition, this survey is intended to be used as an academic analysis only and will not harm anyone.

Thank you for your cooperation and support. Have a great day!

**SECTION 1**	**GENERAL INFORMATION**
The items below are to get basic information about the respondent.<br>Please tick (√) accordingly into the given box (□).	
1. Age	□ 20-29 □ 30-39 □ 40-49<br>□ Over 50 years old
2. Title	□ Assistant Engineer □ Mid-level Engineer<br>□ Senior Engineer □ Other
3. Education Level	□ High school and below □ Bachelor<br>□ Master □ Doctor
4. Work Experience (year)	□ 1-5 □5-10 □10-15 □ More than 15
5. Working Sector	□ Government □ Supervisory Authority<br>□ Construction Company □ Labor Team<br>□ Supplier

**SECTION 2**	**FACTORS AFFECT THE SAFETY OF ASSEMBLY CONSTRUCTION**

Tips:

1. The following questions are intended to investigate possible factors affecting the safety of assembly construction in China.
2. Please carefully read each item in the survey and tick (√) the corresponding scale according to the current situation of your project experience. Each scale represents the following meaning:

1	2	3	4	5	6	7
Very low	low	somewhat low,	medium	somewhat high	high	very high

3. Please answer all the questions and try to make an accurate estimate.

Government department Factors (GD)								
ID	Item	1	2	3	4	5	6	7
G1	Inadequate approval of construction procedures.							
G2	Laxity in enterprise qualification audit.							
G3	Large machinery reporting management.							
G4	Regular supervision is a formality.							
Supervisory authority Factors (SA)								
ID	Item	1	2	3	4	5	6	7
SA1	Blindly approving the wrong safety measures.							
SA2	Confused site supervision system.							
SA3	Failure to conduct on-site supervision.							
Construction company Factors (CC)								
ID	Item	1	2	3	4	5	6	7
C1	Inadequate technical safety briefings.							
C2	Chaotic arrangement of large machinery on-site.							
C3	Lack of on-site safety management.							
C4	Defective construction plans.							
C5	Failure to organize effective safety education and training.							
C6	Poor control of material or equipment supply.							
Site matter Factors (SM)								
ID	Item	1	2	3	4	5	6	7
SM1	Collapse of buildings and or structures.							
SM2	Hit by a robotic arm.							
SM3	Struck by a lifting load.							
SM4	Crane overturning.							
SM5	Environmental damage at the							

	construction site.							
SM6	Electrocution.							
SM7	Worker falls from height.							
Labor team Factors (LC)								
ID	Item	1	2	3	4	5	6	7
L1	Improper driver operation.							
L2	Improper handling by the rigger.							
L3	Signaler command failure.							
L4	Inexperience in construction.							
L5	Poor health status.							
L6	Low safety awareness.							
Supplier Factors (SU)								
ID	Item	1	2	3	4	5	6	7
SU1	Poor quality of wire ropes.							
SU2	Low reliability of hooks and reels.							
SU3	Insufficient load-bearing capacity of wheels or tracks.							
SU4	Brake insensitivity.							
SU5	Unstable foundation.							
SU6	Insufficient operating instructions for machinery.							
SU7	Poor safety climate in the factory.							
SU8	Failure to establish proper installation and dismantling procedures.							
SU9	Poor control of the raw materials used to manufacture the equipment or component.							
